# One of the active ingredients in *Paeoniae Radix Alba* functions as JAK1 inhibitor in rheumatoid arthritis

**DOI:** 10.3389/fphar.2022.906763

**Published:** 2022-09-19

**Authors:** Lu Xiao, Shudian Lin, Feng Zhan

**Affiliations:** Department of Rheumatology and Immunology, Hainan General Hospital (Hainan Affiliated Hospital of Hainan Medical University), Hainan, China

**Keywords:** rheumatoid arthritis, *Paeoniae Radix Alba*, Palbinone, JAK1, network pharmacology

## Abstract

**Objective:** We aimed to explore and verify the mechanism underlying the action of the active ingredients of *Paeoniae Radix Alba* (PRA) in the treatment of rheumatoid arthritis (RA).

**Methods:** The protein targets of PRA’s six active ingredients and RA were identified. Then, the intersection of the two groups was studied. The drug–target network was constructed, visualized, and analyzed by Cytoscape software. Gene Ontology (GO) and Kyoto Encyclopedia of Genes and Genomes (KEGG) enrichment were performed to analyze these genes. Furthermore, we validated our predictions of the potential targets through a docking study. Finally, the anti-inflammatory effect of Palbinone (PB), one of the active ingredients of PRA, was tested by conducting *in vitro* and *in vivo* studies.

**Results:** Six active ingredients of PRA were identified, and 103 overlapping genes were discovered. Functional enrichment analysis indicated that the genes are mostly enriched in IL-17 signaling pathway, Th17 cell differentiation, and the FoxO, ErbB, and TNF signaling pathways. 10 hub genes and two gene cluster modules were identified by Cytoscape. Molecular docking analysis proved that PB was able to bind to the ATP binding site of Janus kinase (JAK)1, thereby acting as a potential inhibitor of JAK1. *In vitro* and *in vivo* studies demonstrated that PB exerts its anti-inflammatory role *via* the inhibition of JAK1.

**Conclusion:** We constructed a multitarget pharmacological network of PRA in RA treatment. PB, one of the active compounds of PRA, was demonstrated to be a promising inhibitor of JAK1.

## 1 Introduction

Rheumatoid arthritis (RA), characterized by synovitis, pannus formation, and bone erosions, is the most common type of chronic autoimmune arthritis (Mohamed Thoufic Ali et al., 2017). RA affects nearly 1% of the world population, carrying huge emotional and financial burden for both the individual and the society (McInnes and Schett, 2017). To date, relieving pain and reducing inflammation by synthetic or biological disease-modifying anti-rheumatic drugs (DMARDs) are the main treatment option for RA (Chen et al., 2019). Although the progression of RA and its detrimental effects on joints could be slowed down with these drugs, a number of patients have inadequate response to treatment. Furthermore, current drug therapies are accompanied by remarkable side effects, including nausea, vomiting, bone marrow suppression, and renal or liver toxicity (Alam et al., 2017). Therefore, recent advancements in medicine and pharmacy have led to the development of more comprehensive and multistage therapies for RA, and alternative medicine has become the patients’ choice. Traditional Chinese medicine (TCM) has become the most frequently used alternative medicine in the prevention and control of RA because of its good therapeutic effect and low toxic side effect (Wang et al., 2019).

*Paeoniae Radix Alba* (PRA), or Bai shao in Chinese, has been used as a medicinal herb in TCM for centuries. PRA is an herbaceous perennial flowering plant in the family of Paeoniaceae. A water/ethanol extract of PRA, known as total glucosides of peony (TGP), is an effective TCM for RA and has been approved as a disease-modifying oral drug since 1998 by the Chinese Food and Drug Administration (Zhang and Dai, 2012; Xiang et al., 2015). Numerous experimental have determined the anti-inflammatory and immunoregulatory actions of TGP in RA treatment, but other active ingredients of PRA that may also play important roles in the immune system have been ignored (Xiang et al., 2015; Huang et al., 2019; Jiang et al., 2020). Therefore, a systematic and comprehensive understanding of the relationships between the targets and pathways of PRA involved in RA treatment is needed.

The rapid progress of bioinformatics, systematic biology, and polypharmacology gave rise to network-based pharmacology as a novel, promising drug development approach. Network pharmacology has been widely applied in many drug discoveries because of its holistic and efficient characteristics for the systematic study of the relationship among drugs, targets, pathways, and diseases. The holistic theory of network pharmacology is also shared by TCM and has long been central to TCM treatments (Ding et al., 2019; Ma et al., 2019; Luo et al., 2020). Thus, network-based pharmacology has become an increasingly valuable technology to explore TCM-related issues.

In this study, network pharmacology was used to elucidate the underlying mechanism of PRA in RA treatment. Firstly, the active ingredients of PRA were identified. Then, the potential molecular targets of PRA were discovered and the intersection of these targets with RA-related proteins were analyzed. Furthermore, we constructed a protein–protein interaction (PPI) network to enlarge the number of proteins that are closely related to the mutual genes. In addition, Gene Ontology (GO) and Kyoto Encyclopedia of Genes and Genomes (KEGG) enrichment of the proteins were performed. Then, docking studies were conducted to verify the chemical force that allowed the active compounds of PRA to bind to their predicted targets and drew the possible binding sites. Finally, the anti-inflammatory effect of Palbinone (PB), one of the active ingredients of PRA, was tested by *in vitro* and *in vivo* studies. Our results may help understand the mechanisms of PRA in RA treatment and facilitate the development of novel drugs.

## 2 Materials and methods

### 2.1 Network pharmacology analysis

#### 2.1.1 Screening of the components and targets of *Paeoniae Radix Alba*

A screening based on absorption, distribution, metabolism, and excretion (ADME) properties (oral availability [OB] ≥ 30%, drug-likeness [DL] ≥ 0.3, 180 Da < molecular weight [MW] < 500 Da, half-life [HL] > 4, AlogP ≤ 5, Hdon ≤ 5) was conducted by the TCM Systems Pharmacology database (TCMSP, http://tcmspw.com/tcmsp.php) to obtain the active compounds of PRA (13). OB represents the percentage of oral medication absorbed into the circulation. DL is used to exclude non-drug-like molecules. HL is defined as the time for the number of drugs in the body to drop by half. Meanwhile, compounds with MW of 180–500 Da are thought to be easier to act as drugs (Lipinski et al., 2001).

**FIGURE 1:**
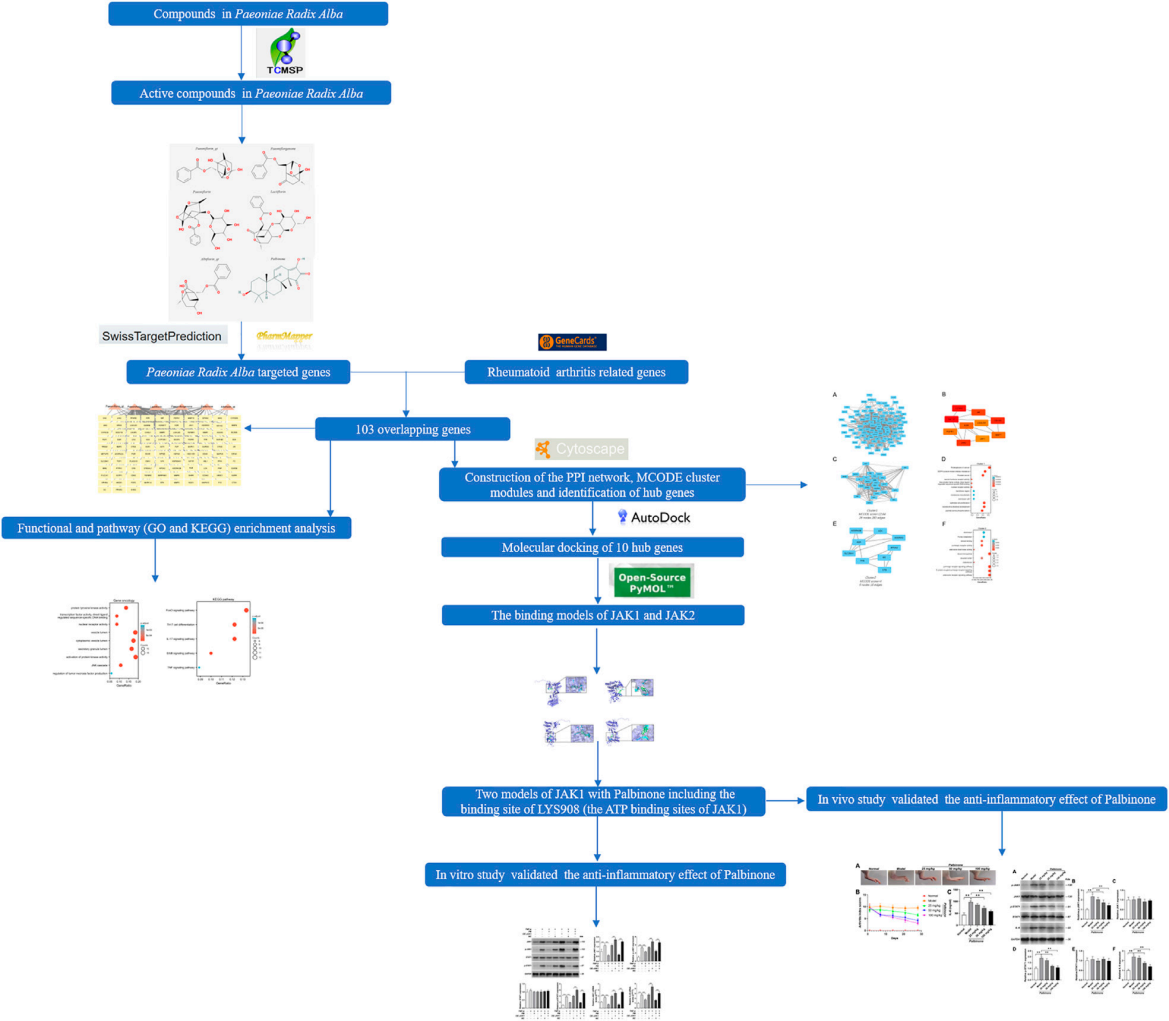
Flowchart of the study.

The chemical structures of the screened components of PRA were searched on PubChem. The active compounds with chemical structures available in the website were subjected to target prediction using different databases (SwissTargetPrediction and PharmMapper) according to the results of the chemical structures (Wang et al., 2017; Wishart et al., 2018). The species was limited to “*Homo sapiens*.” The overall flowchart of this study is shown in Figure 1.

#### 2.1.2 Collection of RA-related genes

RA-related target genes were identified by retrieving public database GeneCards (http://www.genecards.org/) using the keywords, “rheumatoid arthritis” and “*Homo sapiens*.”

#### 2.1.3 Identification of overlapping genes

The overlapping genes between the drug targets and RA were identified and visualized by Venn diagram (http://bioinformatics.psb.ugent.be/webtools/Venn/). The symbols and compounds names of the overlapping genes were uploaded into the Cytoscape (3.8.0) software (Otasek et al., 2019). A network was built to show the relationship between the active ingredients and targeted genes.

#### 2.1.4 Functional and pathway enrichment analysis

R packages (clusterProfile and ggplot2) were used to perform GO enrichment and KEGG pathway analyses for the overlapping genes (Yu et al., 2012). ClusterProfile package was used to analyze, and ggplot2 was applied to visualize the results.

#### 2.1.5 Analysis of protein–protein interaction network and identification of hub genes

A PPI network of the overlapping genes was constructed using the Search Tool for the Retrieval of Interacting Genes/Proteins database (STRING, http://www.string-db.org/) to further investigate the hub genes in PRA for RA treatment (the cut-off standard as a combined score > 0.4). Then, CytoScape software was used to visualize the result. Molecular Complex Detection (MCODE) V1.5.1, which is a plug-in of CytoScape, was used in identifying significant modules (MCODE score ≥ 4) (Bandettini et al., 2012). In addition, CytoHubba, which is another plug-in of Cytoscape, was employed to study essential nodes in the network. Nodes with higher degrees of interaction were considered hub genes (Chin et al., 2014).

#### 2.1.6 Molecular docking

The structures of the active compounds identified in PRA and the 3D crystal structures of the potential target protein were searched from the database of the Research Collaboratory for Structural Bioinformatics Protein Data Bank (PDB, http://www1.rcsb.org/). AutoDock 4.2 software was used to modify the structure and perform molecular docking (Rizvi et al., 2013). The binding energy and binding sites calculated by AutoDock were recorded, and the predicted models were saved in PDB file format. The PyMOL 3.6 software was used to visualize the models.

### 2.2 *In vitro* and *in vivo* study

#### 2.2.1 Cell and reagents

Human Immortalized Synovial Fibroblasts cell line (HISF cell line, iCell-008a) and primary fibroblast basal medium was purchased from iCell Co. (Shanghai, China). The cell line carries the SV40 gene by lentivirus transfection. Immunofluorescence staining of Fibronectin or Vimentin was positive, and the cell purity was higher than 90%. PB (139954-00-0, purity ≥98%) was obtained from ChemFaces Co. (Wuhan, China). Fetal bovine serum (SH30070.03) was purchased from Hyclone, Logan (UT, United States). Tumor necrosis factor (TNF)-alpha (P1001), a recombinant human protein, was purchased from APExBIO Co. (Houston, United States). Primary antibody against p-JAK1 (44-422G) was purchased from Thermo Fisher Scientific Co. (Pittsburgh, PA, United States). Primary antibodies against STAT1 (9175) and p-STAT1 (9167) were purchased from Cell Signaling Technology (Boston, United States). Primary antibodies against JAK1 (ab133666) and GAPDH (ab8245) were purchased from Abcam Co. (Cambridge, United Kingdom). Goat Anti-Rabbit IgG H&L (HRP, ab6721) and Rabbit Anti-Mouse IgG H&L (HRP, ab6728) were also purchased from Abcam Co. (Cambridge, United Kingdom). TRIzol reagent was purchased from Invitrogen (Carlsbad, CA, United States). QuantiTect Reverse Transcription kit was purchased from Qiagen (Valencia, CA, United States). Immunization Grade Bovine Type II Collagen (20022) was obtained from Chondrex (Woodinville, United States). Complete Freund’s adjuvant (F5881) was got from Sigma (St. Louis, MO, United States). Rat IL-6 ELISA Kit (PI328) was purchased from Beyotime (ShangHai, China).

#### 2.2.2 Cell culture and transfection

HISF cells were cultured in primary fibroblast basal medium containing penicillin (final concentration of 100 U/ml), streptomycin (final concentration of 100 μg/ml), primary fibroblast culture additive and 10% FBS in a humidified incubator with 5% CO_2_ at 37°C. The Plvx-puro plasmid vector were obtained from Addgene Co. (Watertown, Massachusetts, United States). The PHelper 1.0, PHelper 2.0 assisted plasmid, and the transfection reagent were purchased from GENE (ShangHai, China). The JAK1 lentivirus and control lentivirus were transfected into 293 T-cells for 48 h. Then, the JAK1 lentivirus supernatant was collected. HISF cells (0.5 × 105 cells per well) were plated into 24 well plates overnight at 37°C. Then, cells were transfected with control lentivirus and JAK1 lentivirus supernatant for 48 h.

#### 2.2.3 Animal experiment

Male Wistar rats (200–250 g) were purchased from Cavens Laboratory Animal Co., Ltd. (http://www.cavens.com.cn/; Changzhou, Jiangsu, China). Rats were kept in cages in a standard environment with a light/dark cycle of 12 h at 21°C and 55% humidity and were permitted to access water and food freely. Rats were randomly separated into five groups (*N* = 6), including normal, collagen-induced arthritis (CIA) model, low concentration (25 mg/kg/day) PB + CIA rats, middle concentration (50 mg/kg/day) PB + CIA rats, and high concentration (100 mg/kg/day) PB + CIA rats, respectively. CIA model was induced according to a published protocol (Brand et al., 2007). Briefly, bovine type II collagen (Chondrex, Redmond, WA, United States) was dissolved in 0.1 M acetic acid overnight at 4 °C. This was emulsified in an equal volume of complete Freund’s adjuvant (Chondrex, Redmond, WA, United States). In order to generate CIA model rats, the rats were immunized intradermally at the base of the tail with 0.1 ml of emulsion containing 100 μg of type II collagen. The rats in the control group were injected with the same volume of sterile normal saline. The second immunization was performed on the 7th day after primary immunization. Arthritis index (AI) was used to evaluate the ankle of model, and AI > 4 was regarded as the model was successfully established. After the establishment of the CIA rat model, PB was administrated every day at indicated concentrations for 28 days. The ankles of the rats were observed on day 1, 14, 21 and 28, and the AI was calculated 24 h after the last administration. AI scoring criteria: 0, normal; 1, erythema, and slight swelling of the ankle joint; 2, erythema and slight swelling of ankle to metatarsal or metacarpal joints; 3, erythema, and moderate swelling from ankle to metatarsophalangeal joint; 4, erythema and severe swelling of the ankle to toe joints.

#### 2.2.4 Western blot assay

Total protein from HISF cells and synovium were extracted with RIPA lysis buffer. Protein was quantified by BCA method. The samples were added to ×5 loading buffer and submerged in the boiling water for 10 min. Semiquantitative immunoblotting was carried out as follows. According to the molecular weight of proteins to be measured, 10% or 12% separation gel and 5% compression gel were prepared. Proteins were separated on polyacrylamide gels by SDS-PAGE at 80 V stable pressure electrophoresis for about 30 min. When the sample was placed in the separation gel, the voltage was adjusted to 120 V, and electrophoresis was continued until the target band reached an appropriate position. Then, the separated protein was transferred to polyvinylidene difluoride (nitrocellulose) membrane in Tris-glycine buffer. After removing the bubble, the membrane was dyed with Ponceau-S for 5 min and then cleaned twice with Tris-buffered saline/Tween 20 (TBST) to observe the protein on the membrane. Then, membranes were blocked with 5% fat-free milk blocking buffer for 2 h at room temperature and then incubated overnight at 4°C with respective antibodies at different working dilutions. After that, membranes were washed thrice with TBST buffer and exposed to horseradish peroxidase-conjugated secondary antibodies for 1 h at room temperature. Then, the membrane was fully exposed to equal volumes of mixed chemiluminescent reagents A and B for 5 min. Tanon 6600 luminescent imaging workstation was used for detection. When the same PVDF film needed to be exposed more than once, a strip solution was used for washing. Figures showed the representative results from experiments repeated at least thrice.

#### 2.2.5 Real time quantitative PCR

The mRNA expressions of JAK1and interleukin-6 (IL-6) were quantified by using qPCR. Based on the instructions of the manufacturer, total RNA of JAK1 and IL-6 was isolated from HISF cells by TRIzol reagent. The system (20 µl) was used to synthesize cDNA. In addition, qPCR was performed using the 20 μl system, which included cDNA (1 μl), nuclease-free water 7.4 μl, Ssofast EvaGreen Supermix (10 μl), forward primer (0.8 μl), and reverse primer (0.8 μl). Fold change (2^−ΔΔCt^) was used to analyze the relative expression of target genes (Pfaffl, 2001). GAPDH was used as the inner standard to normalize the gene concentration of target mRNAs. Each gene analysis was performed in triplicate. The qPCR primers are shown in Supplementary Table S1.

#### 2.2.6 Enzyme-linked immunosorbent assay

ELISA was performed using the IL-6 ELISA kit to identify the level of IL-6 in synovium tissue of indicated groups. The supernatant of synovium tissue is used. Synovial tissue was homogenized with 1.5 ml normal saline in an ice bath, the homogenate was centrifuged at 2,000 RPM for 10 min, and the supernatant was taken for ELISA testing.

#### 2.2.7 Statistical analysis

All experiments were performed independently at least thrice. Image Pro Plus 6.0 software was used to analyze the Western blot results. The results were expressed as mean ± SD. Data were analyzed and plotted by GraphPad Prism 5 (Version 5.01), and collated by Adobe Illustrator CS6 (Version 16.0.0). The statistical significance between different groups was assessed by one-way ANOVA and Tukey’s test. *p* value < 0.05 was considered statistically significant.

## 3 Results

### 3.1 Active ingredients and targets of *Paeoniae Radix Alba*

**FIGURE 2:**
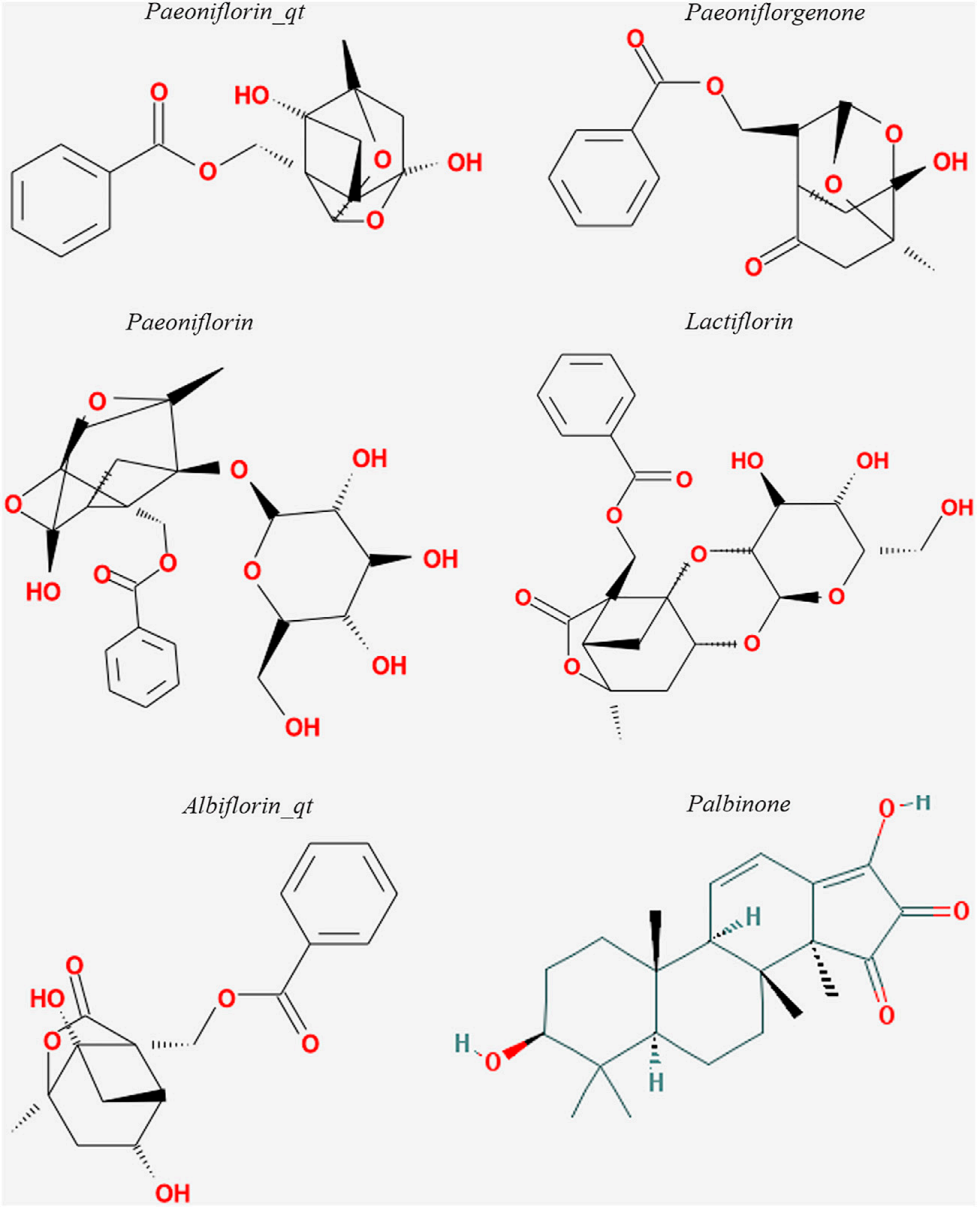
Structures of the six active compounds in *Paeoniae Radix Alba.*

The six active ingredients of PRA, including PB, paeoniflorin_qt, albiflorin_qt, paeoniflorgenone, lactiflorin, and paeoniflorin, were extracted through ADME screening (Supplementary Table S2). The targets of the six compounds were predicted using databases according to 2D and 3D chemical structures (Figure 2). Genes predicted by PharmMapper with Norm Fit ≥0.5 and genes predicted by SwissTargetPrediction with Probability >0 were regarded as drug-targeted genes. In total, 161 and 78 genes were screened in PharmMapper and SwissTargetPrediction, respectively, and 163 genes were screened as drug-targeted genes.

### 3.2 Acquisition of the disease target of rheumatoid arthritis

**FIGURE 3:**
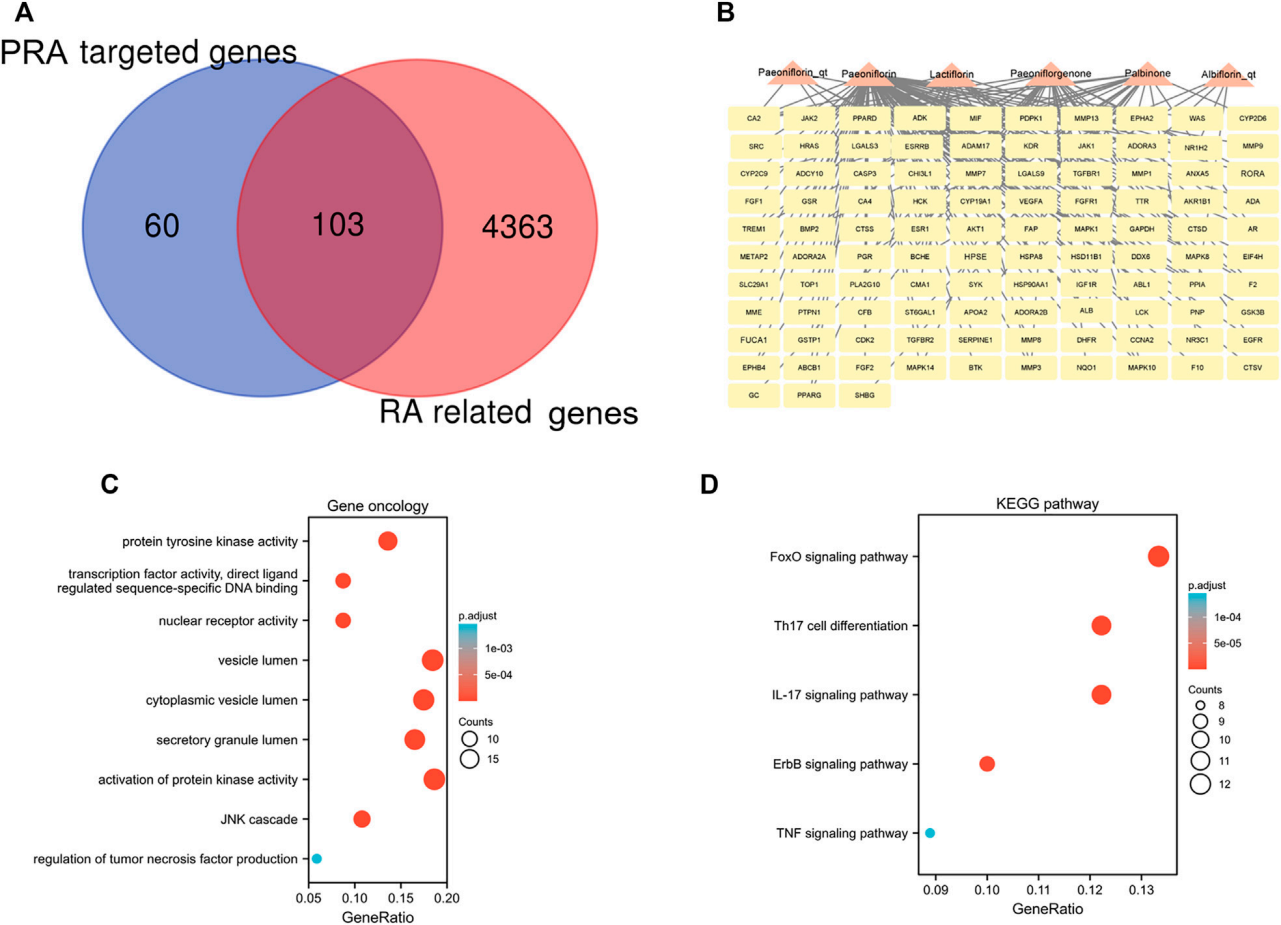
103 intersected genes between *Paeoniae Radix Alba* (PRA) and rheumatoid arthritis (RA) and the functional enrichment of these genes. **(A)** Venn diagram of genes between RA and PRA; **(B)** 103 predicted target genes of PRA (Palbinone, paeoniflorin_qt, albiflorin_qt, paeoniflorgenone, lactiflorin, and paeoniflorin); The enrichment analysis results of **(C)** GO and **(D)** KEGG pathway. Adjusted *p* value < 0.05 was considered significant.

A total of 4,466 human genes associated with RA were collected from databases. Among these genes, 103 were also predicted as the targets of the six active compounds of PRA (Figure 3A; Supplementary Table S3). In addition, a drug-target network was constructed by CytoScape software and is shown in Figure 3B.

### 3.3 Functional and pathway enrichment analyses

The 103 overlapping genes were analyzed using GO and KEGG enrichment (Figures 3C,D). Based on GO enrichment, the biological process acts primarily on activation of protein kinase activity JNK cascade, and regulation of tumor necrosis factor production. These proteins are primarily located in vesicle lumen, cytoplasmic vesicle lumen, and secretory granule lumen. With regard to molecular functions, these proteins play a role in protein tyrosine kinase activity, nuclear receptor activity, and transcription factor activity, direct ligand regulated sequence-specific DNA binding (Figure 3C). KEGG pathway analysis presents that these proteins are primarily involved in IL-17 signaling pathway, Th17 cell differentiation, and the FoxO, ErbB, and TNF signaling pathways (Figure 3D).

### 3.4 Protein–protein interaction network construction, biological functions analyses, and molecular complex detection cluster module identification

**FIGURE 4:**
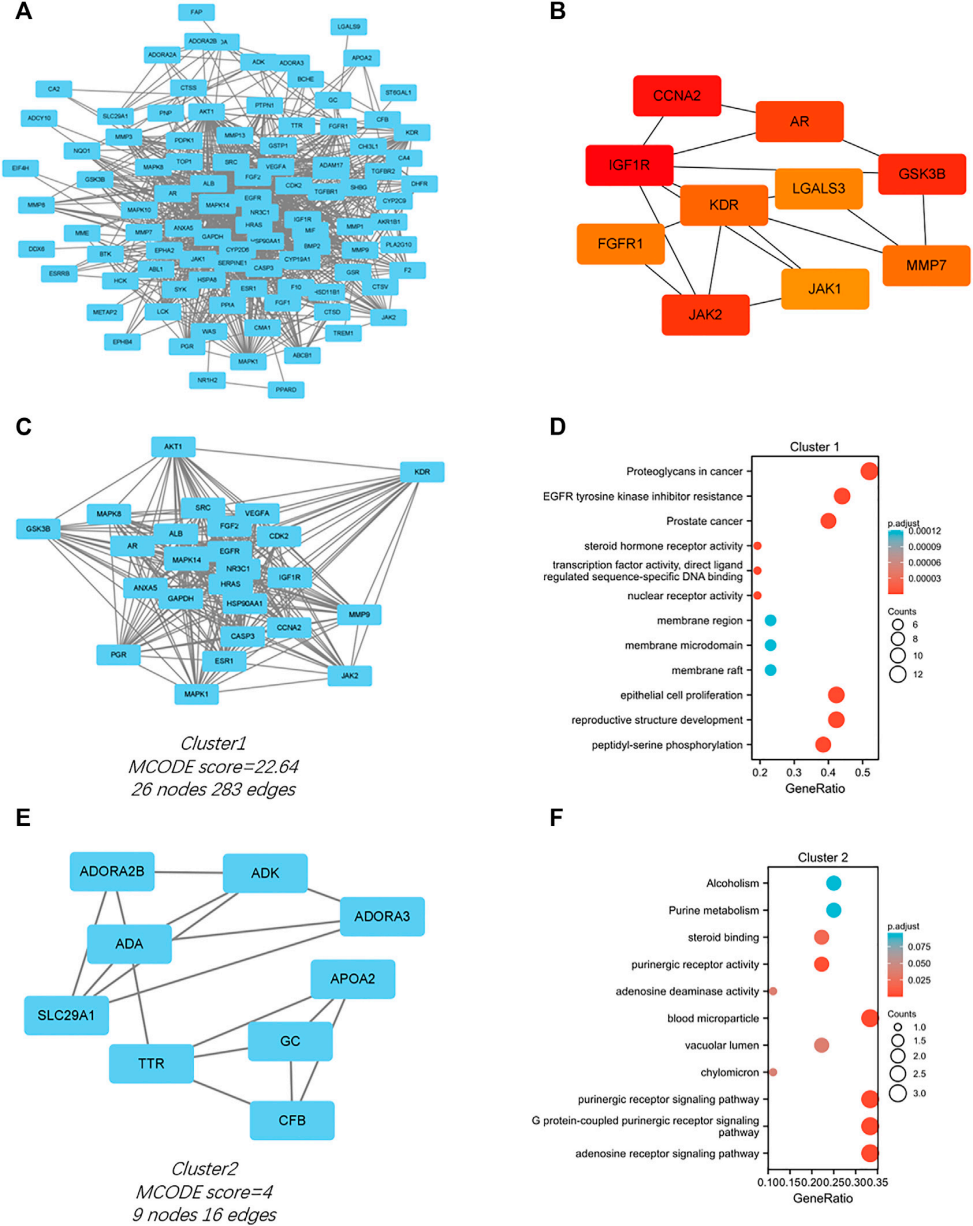
PPI network of the intersected 103 mutual genes, the top 10 identified hub gene web, and cluster modules extracted by MCODE. **(A)** PPI network of the 143 intersected genes; **(B)** a network of top 10 hub genes from the PPI network; Two cluster modules extracted by MCODE. Cluster 1 **(C)** had higher cluster score (MCODE score = 22.64), followed by cluster 2 **(E)** (MCODE score = 4). GO and KEGG enrichment analysis of the modular genes in cluster 1 **(D)** and cluster 2 **(F)**. Adjusted *p* value < 0.05 was considered significant.

The PPI network for the 103 genes was constructed after the common genes were imported to STRING (Figure 4A). The hub genes were calculated using the different algorithms of the plug-in CytoHubba. The top 10 hub genes were screened using the following conditions: ranking according to DMNC score and Degree >20 (median). The top 10 hub genes included insulin-like growth factor 1 receptor (IGF1R), cyclin-A2 (CCNA2), glycogen synthase kinase-3 beta (GSK3B), Janus kinase 2 (JAK2), androgen receptor (AR), kinase insert domain receptor (KDR), matrix metallopeptidase 7 (MMP7), fibroblast growth factor receptor 1 (FGFR1), galectin 3 (LGALS3), and Janus kinase 1 (JAK1, Figure 4B).

Significant modules of the PPI network were identified by MCODE. The MCODE score of 4 was set as the threshold. Two modules had an MCODE score of ≥4, as illustrated in Figures 4C,E. Cluster 1 (MCODE score = 22.64) had 26 nodes and 283 edges (Figure 4C). GO analysis showed that the proteins in cluster 1 were related to peptidyl-serine phosphorylation, reproductive structure development, and epithelial cell proliferation. KEGG pathway analysis showed that these proteins were primarily involved in EGFR tyrosine kinase inhibitor resistance, proteoglycans in cancer, and prostate cancer (Figure 4D). Cluster 2 (MCODE score = 4) had nine nodes and 16 edges (Figure 4E). GO analysis showed that the proteins in cluster 2 were related to adenosine receptor signaling pathway, G protein-coupled purinergic receptor signaling pathway, and purinergic receptor signaling pathway. KEGG pathway analysis showed that these proteins were primarily involved in purine metabolism and alcoholism (Figure 4F).

### 3.5 Molecular docking

**FIGURE 5:**
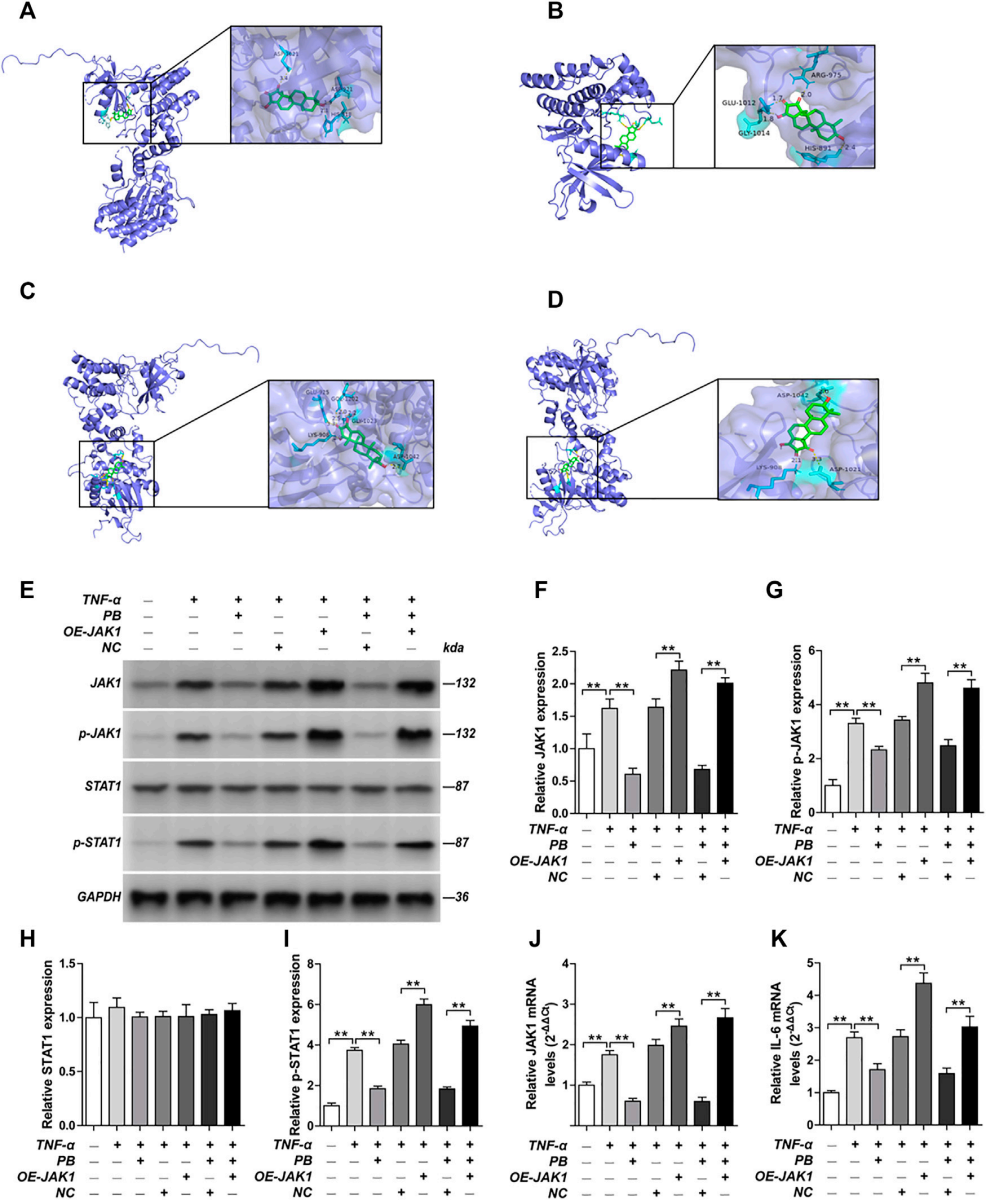
Molecular models of Palbinone (PB) binding to its predicted protein targets and the anti-inflammatory effect of PB by inhibiting JAK1 in HISF cells. **(A)** JAK1 binding PB; **(B)** JAK2 binding PB; **(C)** PB binding to JAK1 in ATP binding sites LYS908; **(D)** PB binding to JAK1 in ATP binding sites LYS908. HISF cells were transfected with NC, OE-JAK1 by lentiviral vector. After HISF cells were treated with TNF-α (20 ng/ml) for 24 h, they were incubated with PB (10 μg/ml) for 6 h. The expression of JAK1 **(E,F)**, p-JAK1 **(E,G)**, STAT1 **(E,H)** and p-STAT1 **(E,I)** from the indicated group were detected by Western blot assay. The mRNA levels of JAK1 **(J)** and IL-6 **(K)** from the indicated group were detected by qPCR. Results were mean ± SD for three individual experiments. *p < 0.05, **p < 0.01.

The PPI network of the 103 overlapping genes was constructed. The top 10 hub genes are shown in Table 1, and the interactions are demonstrated in Figure 4B. The top 10 candidate targets of the six active ingredients of PRA were subjected to molecular docking analysis, and the result provided a visual explanation of the interaction between the active ingredients and their potential targets (Table 1). Janus Kinase (JAK)1 and JAK2, both targeted by PB, have lower binding energies than the other targets; thus, they have higher probabilities to bind. The models of the binding of JAK1 and JAK2 with PB were shown in Figures 5A,B. The AutoDock software predicted 10 binding sites and ranked these sites by binding energy. The predicted binding energy and sites of JAK1 and JAK2 are listed in Table 2. Lysine (LYS) 908 is considered JAK1’s binding site with adenosine-triphosphate (ATP) and mediates the main function of JAK1. Two predicted models of JAK1 with PB including the binding site of LYS908 are exhibited in Figures 5C,D.

**TABLE 1:** The top 10 hub gene targets of *Paeoniae Radix Alba* in RA treatment.

No	Target	Compounds	Binding energy (kcal/mol)	Amino acid in the bond(s)
1	IGF1R	Paeoniflorin	−3.15	ARG1064
2	CCNA2	Paeoniflorgenone	−6.11	TYR179
3	GSK3B	Paeoniflorin	−4.35	GLN89; ARG96
4	JAK2	Palbinone	−7.55	GLU1012; GLY1014; ARG975; HIS891
5	AR	Paeoniflorin	−5.17	ARG752; THR755; ASN756
		Paeoniflorgenone	−7.16	—
6	KDR	Paeoniflorin	−4.57	THR924
7	MMP7	Paeoniflorin	−5.22	—
8	FGFR1	Paeoniflorin	−3.42	GLN491
9	LGALS3	Paeoniflorin	−4.79	GLN187; HIS217; GLN220
10	JAK1	Palbinone	−7.23	HIS918; ASP921; ASP1021

**TABLE 2:** The binding energy and sites of JAK1 and JAK2 with Palbinone ranked by binding energy from Autodock.

	JAK1		JAK2	
	Binding energy (kcal/mol)	Amino acid in the bond(s)	Binding energy (kcal/mol)	Amino acid in the bond(s)
1	−7.23	HIS918; ASP921; ASP1021	−7.55	GLU1012; GLY1014; ARG975; HIS891
2	−7.15	GLY1023; GOL1202; LYS908	−6.94	GLU965; ASN1111
3	−6.7	—	−6.94	GLU1006
4	−6.64	—	−6.84	ARG1113
5	−6.35	ASP921	−6.8	ARG1113
6	−6.14	ASP1024; LYS908	−6.54	ARG980
7	−6.13	—	−6.35	LEU905; TYR913
8	−5.97	ALA1061	−6.31	LEU905; TYR913
9	−5.69	LYS1090; ARG1129	−6.3	GLU877; LYS914
10	−5.31	ILE952; LEU910	−5.86	—

### 3.6 The anti-inflammatory effect of Palbinone by inhibiting JAK1 *in vitro* and *in vivo*

Next, we verified the mechanism underlying the anti-inflammatory effect of PB by *in vitro* and *in vivo* studies. HISF cells were transfected with overexpression-JAK1 (OE-JAK1) by lentiviral vector. After treating the HISF cells with TNF-α (20 ng/ml) for 24 h, they were incubated with PB (10 μg/ml) for 6 h. The transfection efficiency of JAK1 overexpression in HISF cells is shown in Supplementary Figure S1. The protein expressions of JAK1, p-JAK1, signal transducer and activator of transcription (STAT) 1, and p-STAT1 were measured by Western blot. As shown in Figures 5E–I, the notable downregulation of the protein expressions of JAK1, p-JAK1, and p-STAT1 was observed in TNF-α stimulated HSIF cells after PB treatment. Nevertheless, OE-JAK1 obviously inhibited the downregulation of JAK1, p-JAK1, and p-STAT1. In addition, the mRNA levels of JAK1 and IL-6 were detected by qPCR (Figures 5J,K). The expression of IL-6 was decreased significantly after PB treatment, whereas the trend was reversed when JAK1 was overexpressed, indicating that JAK1 is the treatment target of PB during the anti-inflammatory process (Figures 5J,K).

**FIGURE 6:**
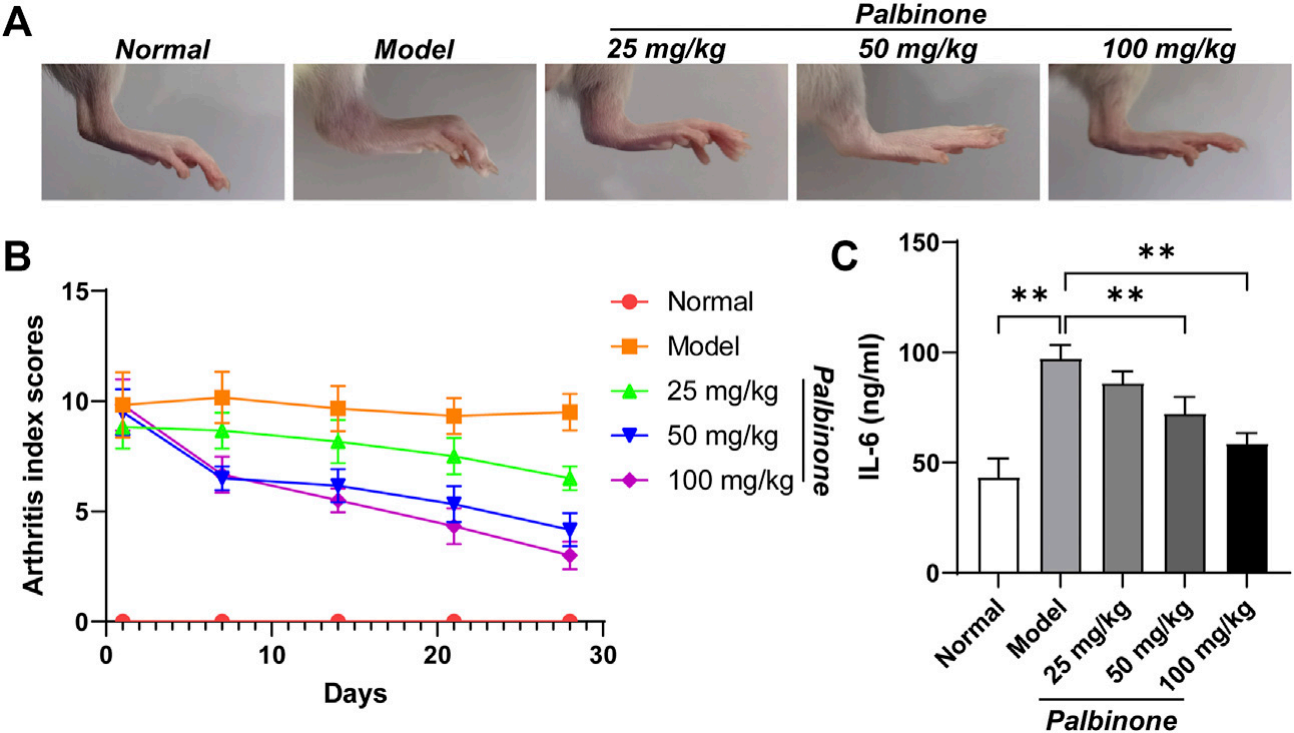
The effects of PB on collagen-induced arthritis rats. Rats were given via intradermal injection of 100 µl of emulsion composed of equal parts of CFA and 2 mg/ml of bovine type II collagen at the base of the tail on day 0 to induce the primary immunization, then they were given the boost immunization consisting of 100 µl of the same emulsion on day 7. Rats were oral gavaged with 25, 50, 100 mg/kg PB for 28 days. The images of rat ankle **(A)**. The arthritis index scores **(B)** were analyzed on day 1, 7, 14, 21, 28 after PB intervention. The expression of IL-6 **(C)** was detected by ELISA. Results were mean ± SD for six individual experiments. *p < 0.05, **p < 0.01.

**FIGURE 7:**
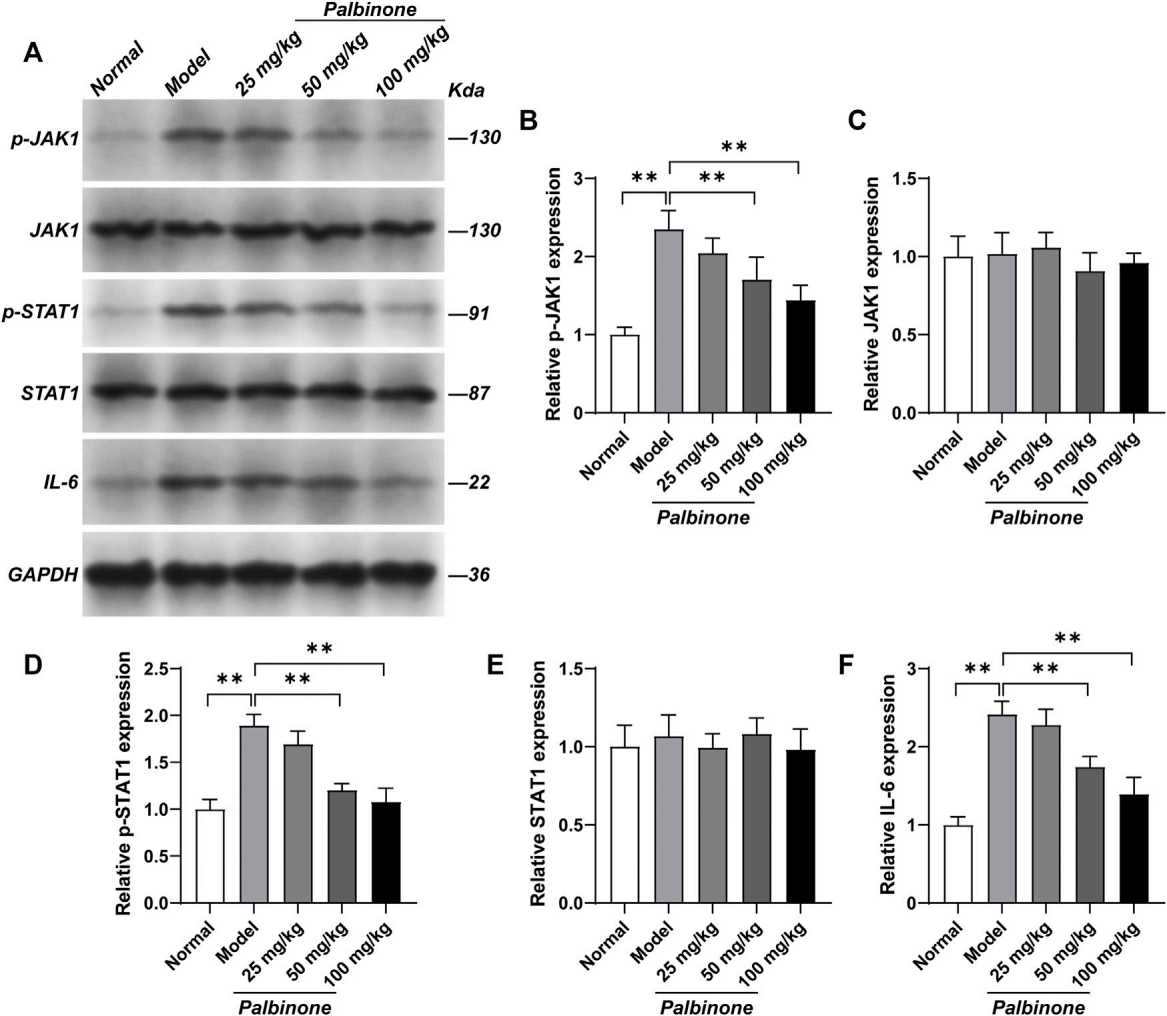
The effects of PB on JAK1-STAT1 signalling pathway in collagen-induced arthritis rats. Rats were given via intradermal injection of 100 µl of emulsion composed of equal parts of CFA and 2 mg/ml of bovine type II collagen at the base of the tail on day 0 to induce the primary immunization, then they were given the boost immunization consisting of 100 µl of the same emulsion on day 7. Rats were oral gavaged with 25, 50, 100 mg/kg PB for 28 days. The expression of p-JAK1 **(A, B)**, JAK1 **(A, C)**, p-STAT1 **(A, D)**, STAT1 **(A, E)** and IL-6 **(A, F)** in synovium were detected by Western Blot. Results were mean ± SD for six individual experiments. *p < 0.05, **p < 0.01.

In addition, *in vivo* study was also performed. Rats were administrated with low, middle, and high concentration of PB. Different concentrations of PB all showed good anti-inflammatory effect and decreased AI (Figures 6A,B) in CIA model rats. Moreover, the level of IL-6 in synovium of CIA model rats has been inhibited by PB (Figure 6C). At protein level, *in vivo* study demonstrated that PB can decrease the protein expressions of p-JAK1 (Figures 7A,B), p-STAT1 (Figures 7A,D), and IL-6 (Figures 7A,F), whereas the total protein of JAK1 (Figures 7A,C), and STAT1 (Figures 7A,E) were not changed.

## 4 Discussion

This research aimed to explore the underlying mechanism of active ingredients in PRA in treating RA. A total of 103 overlapping genes between RA and the drug targets were identified. Functional enrichment analysis indicated that the genes are mostly enriched in IL-17 signaling pathway, Th17 cell differentiation, and the FoxO, ErbB, and TNF signaling pathways. Molecular docking analysis demonstrated that JAK1 and JAK2 may play a central role in the therapeutic process. Meanwhile, PB, one of the active ingredients in PRA, could bind to the ATP binding site of JAK1 and act as a promising inhibitor of JAK1. The anti-inflammatory effect of PB has been verified *in vitro* and *in vivo*.

PRA has shown a rich medicinal value for more than 2000 years. It is used as an effective TCM for autoimmune diseases. TGP, which is a group of glycosides extracted from PRA, is the first immune regulatory drug approved for the treatment of RA in China (Zhang and Wei, 2020). Many studies have focused on discovering the effects of TGP, but research on the role of PRA in RA treatment is rare (Zhao et al., 2018; Li et al., 2019). In our study, we found that PRA may exert therapeutic effects on RA by regulating the IL-17 signaling pathway, Th17 cell differentiation, and the FoxO, ErbB, and TNF signaling pathways. Th17 cells were characterized as a unique Th lineage with distinct secretion of IL-17. Aberrant expression of IL-17 by Th17 cells in RA causes serious autoimmune responses and has recently become a novel research hotspot (Yang et al., 2019). Th17 cell differentiation is affected by numerous positive and negative regulators (Taams, 2020). Th17-related cytokines could activate synovial fibroblasts and macrophages, which are closely related to RA pathogenesis. In 2017, Kim et al. (2017) discovered that interactions between RA fibroblast-like synoviocytes and Th17 cells are involved in the tumorous growth of fibroblast-like synoviocytes and the formation of pannus in joints. The present study indicated that the active compounds in PRA could regulate the IL-17 signaling pathway and Th17 cell differentiation. This regulation may enable PRA to control the abnormal formation of pannus in joints and reduce inflammation in RA. In addition, the FoxO signaling pathway is involved in PRA treatment. This pathway integrates inflammatory stimuli, maintains cell survival in RA synovial tissue, and amplifies inflammatory response (Reedquist et al., 2006). It participates in the cell cycle by targeting different core cycle proteins, such as p27 and p53. The abnormal control of cell cycle in fibroblast-like synoviocytes is a vital pathological process of RA. Fibroblast-like synoviocytes display many features of tumor cells under a chronic inflammatory environment. In due course, these cells eventually escape growth limitations, which induce abnormal proliferation (Mo et al., 2018). Therefore, the effect of PRA on the FoxO signaling pathway may help control the abnormal proliferation of synoviocytes in patients with RA (Dijkers et al., 2000; Cha et al., 2006). In the meantime, we found that the ErbB signaling pathway also participates in the treatment process. The relationship between the ErbB pathway and RA is limited, but this pathway has been widely studied in a variety of cancers. When the pathway is activated, it can trigger a rich network of signaling pathways, such as MAPK, PI3K/AKT, and JAK/STAT, which all take part in RA pathogenesis (Yarden and Sliwkowski, 2001). Therefore, PRA may indirectly affect these pathways by ErbB mediation. In addition, the TNF signaling pathway is also involved. TNF, secreted by activated macrophages and fibroblasts, is a proinflammatory cytokines. TNF is elevated in the synovial fluid, synovial membrane, plasma, and serum of patients with RA (Radner and Aletaha, 2015). Our results suggested that PRA may exert an underlying effect on the TNF signaling pathway. TNF is a key cytokine in RA pathogenesis; thus, PRA may have great potential in RA treatment by regulating this pathway (Yamanaka, 2015).

Docking analysis was performed to verify the chemical force that allows the six active ingredients of PRA (paeoniflorgenone, PB, lactiflorin, paeoniflorin, paeoniflorin_qt, and albiflorin_qt) to bind to the predicted targets. Our research discovered that JAK1 and JAK2 are the vital targets. PB, one of the active compounds of PRA, could bind to the ATP-binding site of JAK1 and act as an inhibitor of JAK1. *In vitro* and *in vivo* studies proved this prediction and demonstrated that PB’s anti-inflammatory effect was *via* the inhibition of JAK1. JAKs interact with various cytokine receptors and couple cytokine binding to cytoplasmic signaling cascades (Hammarén et al., 2015). Its role in the pathological process of RA has been confirmed and attracted research interest (O'Shea et al., 2015). When ATP binds to the ATP-binding site in JAKs and releases its phosphate, the phosphate can then be used by JAKs and cytokine receptors. Recently, this mechanism has been used in the development of small molecule drugs that target RA, such as tofacitinib and baricitinib (Villarino et al., 2017). These drugs competitively inhibit the ATP-binding site and kinase activity. In the present study, we discovered a pattern similar to the pharmacological mechanism of these drugs. PB was able to bind to the ATP-binding site of JAK1 in prediction models and exerted anti-inflammatory effect by inhibiting JAK1. Consequently, PB could be a promising inhibitor of JAK1 in clinical practice and deserves to be further studied.

In the interpretation of our results, the following limitations require careful discussion. On the one hand, the six components were selected by subjective threshold. Other compounds in PRA that may play an important role might have been ignored. On the other hand, further experiments, such as those on the pathways, predicted models, and core targets, must be conducted to verify the conclusion drawn in this study.

This study presented several important findings that might have implications in future research. Firstly, our study determined the pharmacological network affected by PRA in RA treatment. Secondly, JAK1 and JAK2 were demonstrated to be the core targets of PRA in RA therapy. Thirdly, PB, as one of the active compounds of PRA, can bind to the ATP-binding site of JAK1 and serve as a new inhibitor for JAK1. Small molecule drugs, such as tofacitinib and baricitinib, have been widely applied in clinical practice. Thus, we believe that PB has great potential in the treatment of RA. These data illustrated the application of network pharmacology in clinical treatment. In addition, this study could also provide guidance for drug development and further scientific drug research.

## Data Availability

The original contributions presented in the study are included in the article/Supplementary Material, further inquiries can be directed to the corresponding author.

## References

[B1] Alam J. Jantan I. Bukhari S. N. A. (2017). Rheumatoid arthritis: Recent advances on its etiology, role of cytokines and pharmacotherapy. Biomed. Pharmacother. 92, 615–633. 10.1016/j.biopha.2017.05.055 28582758

[B2] Bandettini W. P. Kellman P. Mancini C. Booker O. J. Vasu S. Leung S. W. (2012). MultiContrast delayed enhancement (MCODE) improves detection of subendocardial myocardial infarction by late gadolinium enhancement cardiovascular magnetic resonance: A clinical validation study. J. Cardiovasc. Magn. Reson. 14 (1), 83. 10.1186/1532-429x-14-83 23199362 PMC3552709

[B3] Brand D. D. Latham K. A. Rosloniec E. F. (2007). Collagen-induced arthritis. Nat. Protoc. 2 (5), 1269–1275. 10.1038/nprot.2007.173 17546023

[B4] Cha H. S. Rosengren S. Boyle D. L. Firestein G. S. (2006). PUMA regulation and proapoptotic effects in fibroblast-like synoviocytes. Arthritis Rheum. 54 (2), 587–592. 10.1002/art.21631 16447235

[B5] Chen S. J. Lin G. J. Chen J. W. Wang K. C. Tien C. H. Hu C. F. (2019). Immunopathogenic mechanisms and novel immune-modulated therapies in rheumatoid arthritis. Int. J. Mol. Sci. 20 (6), E1332. 10.3390/ijms20061332 30884802 PMC6470801

[B6] Chin C. H. Chen S. H. Wu H. H. Ho C. W. Ko M. T. Lin C. Y. (2014). cytoHubba: identifying hub objects and sub-networks from complex interactome. BMC Syst. Biol. 8, S11. 10.1186/1752-0509-8-s4-s11 25521941 PMC4290687

[B7] Dijkers P. F. Medema R. H. Pals C. Banerji L. Thomas N. S. Lam E. W. (2000). Forkhead transcription factor FKHR-L1 modulates cytokine-dependent transcriptional regulation of p27(KIP1). Mol. Cell. Biol. 20 (24), 9138–9148. 10.1128/mcb.20.24.9138-9148.2000 11094066 PMC102172

[B8] Ding M. Ma W. Wang X. Chen S. Zou S. Wei J. (2019). A network pharmacology integrated pharmacokinetics strategy for uncovering pharmacological mechanism of compounds absorbed into the blood of Dan-Lou tablet on coronary heart disease. J. Ethnopharmacol. 242, 112055. 10.1016/j.jep.2019.112055 31276751

[B9] Hammarén H. M. Ungureanu D. Grisouard J. Skoda R. C. Hubbard S. R. Silvennoinen O. (2015). ATP binding to the pseudokinase domain of JAK2 is critical for pathogenic activation. Proc. Natl. Acad. Sci. U. S. A. 112 (15), 4642–4647. 10.1073/pnas.1423201112 25825724 PMC4403165

[B10] Huang Y. Wang H. Chen Z. Wang Y. Qin K. Huang Y. (2019). Efficacy and safety of total glucosides of paeony combined with methotrexate and leflunomide for active rheumatoid arthritis: A meta-analysis. Drug Des. devel. Ther. 13, 1969–1984. 10.2147/dddt.s207226 PMC6588713 31354242

[B11] Jiang H. Li J. Wang L. Wang S. Nie X. Chen Y. (2020). Total glucosides of paeony: A review of its phytochemistry, role in autoimmune diseases, and mechanisms of action. J. Ethnopharmacol. 258, 112913. 10.1016/j.jep.2020.112913 32371143

[B12] Kim E. K. Kwon J. E. Lee S. Y. Lee E. J. Kim D. S. Moon S. J. (2017). IL-17-mediated mitochondrial dysfunction impairs apoptosis in rheumatoid arthritis synovial fibroblasts through activation of autophagy. Cell Death Dis. 8 (1), e2565. 10.1038/cddis.2016.490 PMC5386390 28102843

[B13] Li B. He S. Liu R. Huang L. Liu G. Wang R. (2019). Total glucosides of paeony attenuates animal psoriasis induced inflammatory response through inhibiting STAT1 and STAT3 phosphorylation. J. Ethnopharmacol. 243, 112121. 10.1016/j.jep.2019.112121 31356966

[B14] Lipinski C. A. Lombardo F. Dominy B. W. Feeney P. J. (2001). Experimental and computational approaches to estimate solubility and permeability in drug discovery and development settings. Adv. Drug Deliv. Rev. 46 (1-3), 3–26. 10.1016/s0169-409x(00)00129-0 11259830

[B15] Luo T. T. Lu Y. Yan S. K. Xiao X. Rong X. L. Guo J. (2020). Network pharmacology in research of Chinese medicine formula: Methodology, application and prospective. Chin. J. Integr. Med. 26 (1), 72–80. 10.1007/s11655-019-3064-0 30941682

[B16] Ma C. Xu T. Sun X. Zhang S. Liu S. Fan S. (2019). Network pharmacology and bioinformatics approach reveals the therapeutic mechanism of action of baicalein in hepatocellular carcinoma. Evid. Based. Complement. Altern. Med. 2019, 7518374. 10.1155/2019/7518374 PMC6390240 30891079

[B17] McInnes I. B. Schett G. (2017). Pathogenetic insights from the treatment of rheumatoid arthritis. Lancet 389 (10086), 2328–2337. 10.1016/s0140-6736(17)31472-1 28612747

[B18] Mo B. Y. Guo X. H. Yang M. R. Liu F. Bi X. Liu Y. (2018). Long non-coding RNA GAPLINC promotes tumor-like biologic behaviors of fibroblast-like synoviocytes as MicroRNA sponging in rheumatoid arthritis patients. Front. Immunol. 9, 702. 10.3389/fimmu.2018.00702 29692777 PMC5902673

[B19] Mohamed Thoufic Ali A. M. Agrawal A. Sajitha Lulu S. Mohana Priya A. Vino S. MohAnA PriyA A. (2017). RAACFDb: Rheumatoid arthritis ayurvedic classical formulations database. J. Ethnopharmacol. 197, 87–91. 10.1016/j.jep.2016.06.047 27329782

[B20] O'Shea J. J. Schwartz D. M. Villarino A. V. Gadina M. McInnes I. B. Laurence A. (2015). The JAK-STAT pathway: Impact on human disease and therapeutic intervention. Annu. Rev. Med. 66, 311–328. 10.1146/annurev-med-051113-024537 25587654 PMC5634336

[B21] Otasek D. Morris J. H. Bouças J. Pico A. R. Demchak B. (2019). Cytoscape automation: Empowering workflow-based network analysis. Genome Biol. 20 (1), 185. 10.1186/s13059-019-1758-4 31477170 PMC6717989

[B22] Pfaffl M. W. (2001). A new mathematical model for relative quantification in real-time RT-PCR. Nucleic Acids Res. 29 (9), e45. 10.1093/nar/29.9.e45 11328886 PMC55695

[B23] Radner H. Aletaha D. (2015). Anti-TNF in rheumatoid arthritis: An overview. Wien. Med. Wochenschr. 165 (1-2), 3–9. 10.1007/s10354-015-0344-y 25651945

[B24] Reedquist K. A. Ludikhuize J. Tak P. P. (2006). Phosphoinositide 3-kinase signalling and FoxO transcription factors in rheumatoid arthritis. Biochem. Soc. Trans. 34 (5), 727–730. 10.1042/bst0340727 17052183

[B25] Rizvi S. M. Shakil S. Haneef M. (2013). A simple click by click protocol to perform docking: AutoDock 4.2 made easy for non-bioinformaticians. Excli J. 12, 831–857. 26648810 PMC4669947

[B26] Taams L. S. (2020). Interleukin-17 in rheumatoid arthritis: Trials and tribulations. J. Exp. Med. 217 (3), e20192048. 10.1084/jem.20192048 32023342 PMC7062523

[B27] Villarino A. V. Kanno Y. O'Shea J. J. (2017). Mechanisms and consequences of Jak-STAT signaling in the immune system. Nat. Immunol. 18 (4), 374–384. 10.1038/ni.3691 28323260 PMC11565648

[B28] Wang Q. Shi G. Zhang Y. Lu F. Xie D. Wen C. (2019). Deciphering the potential pharmaceutical mechanism of GUI-ZHI-FU-LING-WAN on systemic sclerosis based on systems biology approaches. Sci. Rep. 9 (1), 355. 10.1038/s41598-018-36314-2 30674993 PMC6344516

[B29] Wang X. Shen Y. Wang S. Li S. Zhang W. Liu X. (2017). PharmMapper 2017 update: A web server for potential drug target identification with a comprehensive target pharmacophore database. Nucleic Acids Res. 45 (W1), W356–w360. 10.1093/nar/gkx374 28472422 PMC5793840

[B30] Wishart D. S. Feunang Y. D. Guo A. C. Lo E. J. Marcu A. Grant J. R. (2018). DrugBank 5.0: A major update to the DrugBank database for 2018. Nucleic Acids Res. 46 (D1), D1074–d1082. 10.1093/nar/gkx1037 29126136 PMC5753335

[B31] Xiang N. Li X. M. Zhang M. J. Zhao D. B. Zhu P. Zuo X. X. (2015). Total glucosides of paeony can reduce the hepatotoxicity caused by Methotrexate and Leflunomide combination treatment of active rheumatoid arthritis. Int. Immunopharmacol. 28 (1), 802–807. 10.1016/j.intimp.2015.08.008 26292180

[B32] Yamanaka H. (2015). TNF as a target of inflammation in rheumatoid arthritis. Endocr. Metab. Immune Disord. Drug Targets 15 (2), 129–134. 10.2174/1871530315666150316121808 25772178

[B33] Yang P. Qian F. Y. Zhang M. F. Xu A. L. Wang X. Jiang B. P. (2019). Th17 cell pathogenicity and plasticity in rheumatoid arthritis. J. Leukoc. Biol. 106 (6), 1233–1240. 10.1002/jlb.4ru0619-197r 31497905

[B34] Yarden Y. Sliwkowski M. X. (2001). Untangling the ErbB signalling network. Nat. Rev. Mol. Cell Biol. 2 (2), 127–137. 10.1038/35052073 11252954

[B35] Yu G. Wang L. G. Han Y. He Q. Y. (2012). clusterProfiler: an R package for comparing biological themes among gene clusters. Omics 16 (5), 284–287. 10.1089/omi.2011.0118 22455463 PMC3339379

[B36] Zhang L. Wei W. (2020). Anti-inflammatory and immunoregulatory effects of paeoniflorin and total glucosides of paeony. Pharmacol. Ther. 207, 107452. 10.1016/j.pharmthera.2019.107452 31836457

[B37] Zhang W. Dai S. M. (2012). Mechanisms involved in the therapeutic effects of Paeonia lactiflora Pallas in rheumatoid arthritis. Int. Immunopharmacol. 14 (1), 27–31. 10.1016/j.intimp.2012.06.001 22705050

[B38] Zhao Z. Han Y. Zhang Z. Li W. Ji X. Liu X. (2018). Total glucosides of paeony improves the immunomodulatory capacity of MSCs partially via the miR-124/STAT3 pathway in oral lichen planus. Biomed. Pharmacother. 105, 151–158. 10.1016/j.biopha.2018.05.076 29852392

