# Supplementary material for: One of the active ingredients in Paeoniae Radix Alba functions as JAK1 inhibitor in rheumatoid arthritis
Source: Front Pharmacol. 2022 Sep 19;13:906763. doi: 10.3389/fphar.2022.906763 (PMC9527307; doi:10.3389/fphar.2022.906763)
Supplement: Supplementary file 1 [file DataSheet1.PDF]

Supplementary Material

1 Supplementary Data

Supplementary Material should be uploaded separately on submission. Please include any supplementary data, figures and/or tables. All supplementary files are deposited to FigShare for permanent storage and receive a DOI.

Supplementary material is not typeset so please ensure that all information is clearly presented, the appropriate caption is included in the file and not in the manuscript, and that the style conforms to the rest of the article. To avoid discrepancies between the published article and the supplementary material, please do not add the title, author list, affiliations or correspondence in the supplementary files.

2 Supplementary Figures and Tables

For more information on Supplementary Material and for details on the different file types accepted, please see here. Figures, tables, and images will be published under a Creative Commons CC-BY licence and permission must be obtained for use of copyrighted material from other sources (including re-published/adapted/modified/partial figures and images from the internet). It is the responsibility of the authors to acquire the licenses, to follow any citation instructions requested by third-party rights holders, and cover any supplementary charges.

2.1 Supplementary Figures

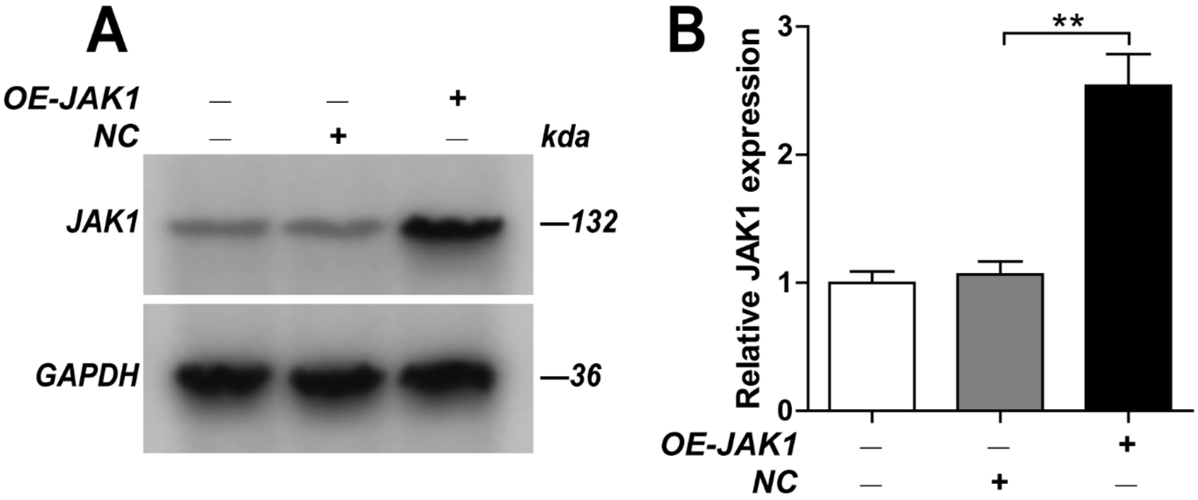

Supplementary Figure 1 Transfection efficiency of JAK1 overexpression in HISF cells verified by Western blot assay.

Results were mean ± SD for three individual experiments. *P<0.05, **P<0.01.

Supplementary tables

Supplementary table 1 Primers used in Quantitative Real-Time PCR

Primers	Sequence (5’→3’)	
IL-6	Forward	AGTTGCCTTCTCCCTGG
	Reverse	ATTCGTTCTGAAGAGGTGAG
JAK1	Forward	ATCGAGCGCACAAAGTTATC
	Reverse	AGCTCCTCATTTTAGCACAG
GAPDH	Forward	AATGAATGGGCAGCCGTTA
	Reverse	TGTAAACCATGTAGTTGAGGT

Supplementary Table 2 Chemical properties of *Paeoniae Radix Alba* active ingredients

MOLID	Molecular name	MW (g/mol)	OB%	DL	AlogP	HL
MOL001919	Palbinone	358.52	43.56	0.53	2.69	4.34
MOL001925	Paeoniflorin_qt	318.35	68.18	0.40	0.46	8.81
MOL001928	Albiflorin_qt	318.35	66.64	0.33	0.42	6.54
MOL001918	Paeoniflorgenone	318.35	87.59	0.37	0.79	7.45
MOL001921	Lactiflorin	462.49	49.12	0.80	-0.57	7.26
MOL001924	Paeoniflorin	480.51	53.87	0.79	-1.28	13.88

Supplementary table 3 the intersection gene of Paeoniae Radix Alba and RA

No	Compound	Gene symbols	Total
1	Paeoniflorgenone	CA2, PPARD, CFB, NR1H2, PDPK1, MAPK1, BCHE, CYP19A1, TREM1, MAPK14, F2, GC, DDX6, MAPK8, EGFR, NQO1, MMP3, PGR, CASP3, RORA, CCNA2, TTR, ADAM17, BMP2, MAPK10, AR, AKR1B1, ESR1, HSD11B1	29
2	Palbinone	CA2, CYP2D6, SHBG, ALB, JAK2, CYP19A1, ESRRB, ADCY10, APOA2, CYP2C9, PGR, CASP3, ST6GAL1, NR3C1, BMP2, JAK1, AR, ESR1	18
3	Lactiflorin	CA2, CFB, PPARG, NR1H2, BCHE, TREM1, PPIA, F2, EPHB4, DDX6, CTSD, MAPK8, CMA1, MMP3, MAPK10	15
4	Paeoniflorin	SYK, PPARD, LGALS3, ALB, GSR, SERPINE1, BCHE, TREM1, PPIA, MAPK14, KDR, HRAS, FGF2, DDX6, MMP7, CTSS, GAPDH, MMP13, TGFBR2, CTSD, LCK, VEGFA, ADORA2A, MAPK8, MME, PTPN1, IGF1R, MMP3, F10, FGFR1, ADORA3, LGALS9, CTSV, ABL1, RORA, FAP, ADK, TTR, ADAM17, MAPK10, AR, AKR1B1, CA2, MMP8, CFB, AKT1, FGF1, GSK3B, PPARG, NR1H2, CA4,PLA2G10, MAPK1, EPHA2, FUCA1, EIF4H, HSPA8, F2, MMP1, EPHB4, ADA, GSTP1, ANXA5, MIF, METAP2, EGFR, SLC29A1, TGFBR1, SRC, CYP2C9, CDK2, ABCB1, DHFR, PGR, PNP, CASP3, HSP90AA1, HPSE, ADORA2B, MMP9, WAS, TOP1, BMP2, ESR1, HSD11B1, BTK	86
5	Paeoniflorin_qt	CA2, NR1H2, CHI3L1, MAPK1, BCHE, PPIA, DDX6	7
6	Albiflorin_qt	HCK, NR1H2, BCHE, PPIA, HSPA8	5
